# Spanish validation of the self-evaluation of negative symptoms scale SNS in an adolescent population

**DOI:** 10.1186/s12888-019-2314-1

**Published:** 2019-10-29

**Authors:** Juan F. Rodríguez-Testal, Salvador Perona-Garcelán, Sonia Dollfus, María Valdés-Díaz, Jesús García-Martínez, Miguel Ruíz-Veguilla, Cristina Senín-Calderón

**Affiliations:** 1 Personality, Evaluation and Psychological Treatment Department, University of Seville, Seville, Spain. Av. Camilo José Cela, 41018 Seville, SN Spain; 2 Virgen del Rocío Outpatient Mental Hospital, University Hospital Virgen del Rocío, Avenue Manuel Siurot, 41013 Seville, SN Spain; 3 CHU de Caen, Service universitaire de Psychiatrie, Centre Esquirol, Avenue Côte de Nacre, F-14000 Caen, France; 4 UNICAEN, UFR Médecine, F-14074 Caen, France; 5 Department of Psychology, University of Cadiz, Avenue República Árabe Saharaui SN. 11510 Puerto Real, Cádiz, Spain

**Keywords:** Negative symptoms, Adolescence, SNS, Psychosis, General population

## Abstract

**Background:**

Negative symptoms (NS) may be observed in the general population in an attenuated form and in high-risk mental states. However, they have been less studied in the general population than positive symptoms, in spite of their importance at the insidious onset of schizophrenia and their appearance before positive symptoms. This study aimed to analyze the empirical structure of the Spanish version of the Self-Evaluation of Negative Symptoms (SNS) Scale and find its psychometric properties and invariance of measurement across sex and age in a sample of adolescents.

**Methods:**

The sample consisted of 4521 adolescents (53.6% female) from 11 to 18 years of age.

**Results:**

Confirmatory Factor Analysis of the SNS confirmed an internal structure of five first-order factors by the characteristic dimensions of NS: avolition, social withdrawal, diminished emotional range, anhedonia, alogia, and one second-order factor which includes the total NS score. Multi-group confirmatory factor analysis showed that the scale was invariant across sex and age. Total scale reliability was adequate. A strong relationship was found between the SNS with depressive symptomatology, moderate with ideas of reference and low with aberrant salience. **Conclusion:** The results back use of the Spanish version of the SNS scale for detection of NS in the general population of adolescents.

## Background

Negative symptoms (NS) are defined as the diminution in or absence of affective-motivational responses typical of adapted functioning [1]. It is generally agreed that NS may be summarized as a diminution or loss of: range/intensity of affective responses (blunted affect), spontaneous speech (alogia), social interest (social withdrawal), interest/maintenance of activities (avolition), enjoyment in carrying out activities (anhedonia) [2–5], and more recently, loss of normal distress has been added [6]. Factor analyses of the NS scales show two differentiated factors: diminished expression (expressive), and avolition/apathy/amotivation (experiential) [7–9].

NS are salient in the insidious onset of schizophrenia, and are major in one third to half of first episodes [10], appearing before positive symptoms [11], and predicting them [12]. Although NS are observed in at least 50% of persons with schizophrenia [3] and 70% before a psychotic episode [13], they are not exclusive to it, but are also important in affective and cognitive disorders [14]. In the schizophrenia spectrum, they are more frequent, long-lasting, and avolition/apathy/amotivation is more prominent than the expressive component. Outside of this spectrum, they tend to be transitory and secondary [15].

As NS tend to precede positive symptoms and the onset of psychotic decompensation, it would have to be wondered whether it is possible to observe NS in the general population in order to improve early identification of psychosis. This would imply that NS represent Psychotic-Like Experiences (PLE), in the sense that is usually applied to subthreshold positive symptoms, and would then be considered on a continuum from healthy functioning to frank syndromes [16].

Although the nearly complete absence of NS in the general population has often been mentioned, in analyses of large populations, somewhat over 20% of the participants have been observed to show at least one NS [17]. There is therefore evidence that NS are expressed, at least in an attenuated form, in the general population like PLEs [18–20].

However, it has been said that while positive indicators fit well to a dimensional perspective, NS may be represented better from a categorical perspective [21]. In general, a quasi-continuum relationship between the general population and psychosis is alluded to when positive and negative symptoms and other indicators characteristic of the onset of psychosis are approached [22, 23].

Since the first psychotic episode usually occurs between 15 and 30 years of age, adolescence is a critical period for study of NS [24]. Adolescent PLEs show a variety of patterns: paranoid thoughts and/or hallucinations, and isolated NS, in addition to anxiety and depression as risk factors. Observed experiences corroborated in the general population include diminished range of expression and anhedonia dimensions, for which a large proportion of adolescents do not request clinical help [25].

As PLEs are weak positive predictors of the transition to psychosis, subthreshold NS may be better predictors of its onset [26], and their study in an adolescent population could be important, even among those who have not yet requested clinical help [27]. Calkins et al. [28] verified the persistence or worsening of 51% of the baseline indicators at a two-year follow-up of community youths, for which positive indicators had a predictive value of 0.51 and negative of 0.83. These authors emphasized that youths with persistent symptomatology do not always seek help, which is relevant for early detection of psychosis and its prognosis.

Therefore, transfer of the study of NS to the adolescent evolutionary period is of interest. Considering the combination of positive and negative indicators, their persistence and severity, their study in the general population is important to progress in the study of PLE or attenuated psychotic symptoms [29].

Attention has been given NS in studies of youths with at-risk mental states, due to the low predictive power of traditional UHR (Ultra High Risk) criteria for psychosis. Moderate to severe NS are often found in adolescents at clinical risk [30, 31], where persistence is the best predictor of transition to psychosis, ahead of severity [32, 33], and presence of positive [34] and disorganized [35] symptoms.

Of the NS which best predict the transition to psychosis with UHR criteria, the best are blunted affect [36], and anhedonia [37–40], indicators which are maintained in those who do not transition [41].

However, it is not sufficiently clear whether NS with UHR criteria are predictors only of developing psychosis, or also of other severe psychopathologies such as emotional disorders [42]. For example, the importance of mood alteration has been emphasized in clinical trajectories of UHR [43]. It has also been suggested that some NS, such as avolition, may have to do with anxiety because of the first psychotic symptoms, as a way of protecting self-esteem [44], which would explain symptoms of anxiety and depression among those who do not transition to psychosis.

Some recent results specify, however, that anhedonia in UHR participants who transitioned to psychosis, is independent of concurrent positive and depressive symptoms (including suicidal ideation) [45]. This emphasizes anhedonia as an early risk characteristic for psychosis, and more related to severity of NS than to depression.

In view of all of the above, the analysis and follow-up of NS as early prodromal symptoms in adolescence is relevant. The NS become more frequent and their severity fluctuates when the psychotic episode has developed than in the UHR state itself [39, 44], increasing the risk of psychosis and worsening the prognosis in UHR [46].

Whether like PLE (transitory) or defined prodromal indicators (clearly stable), it is advisable to have evaluation instruments which can identify NS in adolescence; an initial screening that facilitates characterization of these manifestations, and presumably, sheds light on the processes prior to onset of psychosis. However, there are not many self-report instruments evaluating the different dimensions of NS. A review by Lincoln, Dollfus and Lyne [47] highlighted 12 scales evaluating NS, but only three are exclusively for NS. Of the other nine, six evaluate subdomains of NS and three are psychopathological scales with some NS. Of these 12 scales, four are adapted to Spanish, but none of them is specific to evaluation of NS.

The SNS scale [11] is to date the most complete, as well as the briefest, self-report for evaluating the dimensions of NS: social withdrawal, avolition, alogia, anhedonia and diminished emotional range. It has been translated into sixteen languages and has demonstrated adequate psychometric properties of reliability, evidence of convergent, discriminant and construct validity [47].

The general objective of this study was to adapt and validate a Spanish version of the SNS as an NS instrument for application to the general adolescent population. Early identification of NS could be of enormous utility for a symptomatology which may become persistent and erode functioning [11]. The self-report may be more useful and easier to apply in a community population which has not requested medical help. This format can communicate experiential aspects hard for an adolescent to transmit spontaneously, showing as it has with patients with schizophrenia, the validity and reliability of self-reported responses on the symptoms [48].

The specific objectives of this study enabled several precise analyses of NS in adolescents in the general population. These objectives were to: 1) Analyze participants’ responses to each item of the SNS scale and its psychometric characteristics, 2) Study the factor structure of the SNS scale, comparing its structure to the one found in other studies for adult and patient populations, 3) Analyze the scale’s invariance of measurement across sex and age, 4) Study the psychometric properties of the SNS scale, its reliability and convergent and divergent validity, for use in the general population, and 5) Find the cutoff point of the SNS scale, its sensitivity and specificity in adolescents.

## Method

### Participants

The final sample consisted of 4521 participants (53.6% female) in Western Andalusia (Spain) after exclusion of 137 participants because they were over the age of 18 or had not filled out the tests properly. The average age was 14.32 (*SD* = 1.66, range 11–18 years). The average Hollingshead [49] Social Class Index (SCI) was 44.83 (mean social class, *SD* = 21.48).

### Measures

#### First self-reported evaluation (instrument developed by authors)

This identified the social class index (SCI) [45], current illnesses, psychopathological antecedents, history and duration of symptoms, psychopharmacological treatments and use of other drugs.

#### Self-evaluation of negative symptoms [11]

The scale is comprised of 20 items with three answer choices (0 = “strongly disagree”, 1 = “somewhat agree”, 2 = “strongly agree”). A total score can be found by adding up the answers to all the items. The scale covers five dimensions: avolition, social withdrawal, diminished emotional range, anhedonia, and alogia. Avolition evaluates the lack of motivation, initiative and energy for carrying out different activities as well as maintaining a regular habit. Example item, 15. *There are many things I don’t do because of lack of motivation or because I don’t feel like it*. Social withdrawal refers to the preference for being alone and low need for social contact. Example item 4. *I don’t particularly try to contact and meet friends (letters, telephone, text messaging,* etc.*)*. Diminished emotional range refers to difficulty in experiencing positive and/or negative emotions. Example item, 6. *There are many happy or sad things in life but I don’t feel concerned by them*. The items assessing anhedonia refer to reduced ability to experience pleasure. Example item, 19. *When I imagine doing one thing or another, I don’t feel any particular pleasure in the idea.* Alogia assesses the difficulty for communicating and interacting with others. Example item, 10. *I find it 10 times harder to talk than most people do*.

A factor analysis by the authors of the scale found two factors with patients with schizophrenia and schizoaffective disorder. The first factor contained avolition, asociality, alogia and anhedonia, and the second factor, diminished emotional range. The Cronbach’s alpha found by the authors was .86.

#### The aberrant salience inventory [50]

Spanish version by Fernández-León et al. [51]. This 29-item true-or-false scale is a measure of proneness to psychosis which evaluates assignment of meaning or importance to neutral or irrelevant stimuli. The authors found a Cronbach’s alpha = .89 and adequate convergent and discriminant validity. The Spanish version of the ASI has an *α* of up to .83.

#### Referential thinking scale (REF) [52]

This is a 34-item true-or-false self-report questionnaire on ideas of reference. High scores show overinterpretation of environmental signs and attribution of a special meaning for the subject. The scale has an internal consistency of .83 to .85, retest reliability of .86 (four-week interval), and adequate validity indicators. The Spanish version of the REF scale has an *α* of up to .90 and a retest *α* of .76 (average interval of 44 days in patients [53]).

#### Children’s depression inventory CDI [54]

Spanish version by Del Barrio and Carrasco-Ortiz [55]. This scale comprised of 27 items, which assess depressive symptomatology in children and adolescents, has a three-point Likert-type response (0 = “normality”, 1= “Certain intensity in response” and 2 = “Presence of depressive symptom). The Spanish adaptation has adequate internal consistency (Cronbach’s alpha = .79).

### Procedure

The SNS scale was translated to Spanish. The translation was reviewed and approved by the authors of the scale.

Data were acquired from June 2016 to June 2017 at 29 high schools. Authorization to carry out the study was requested from the schools, and parents were informed of its purpose and requested their written consent authorizing participation. The evaluation tests were administered in group by experienced psychologists in the classrooms at each school.

### Data analysis

A frequency analysis was done of SNS item responses, and skewness and kurtosis were calculated. Exploratory Factor Analyses (EFA) were done of the SNS scale on the polychoric correlations matrix with Robust Diagonally Weighted Least Squares (RDWLS) and Direct Oblimin rotation. A Confirmatory Factor Analysis (CFA) was done to test the suitability of the internal structure with the RDWLS method. Chi squared, Comparative Fit Index (CFI), Goodness of Fit Index (GFI), and the Non-Normed Fit Index (NNFI) which must be >.90 [56] were used to test the overall fit of models. In addition to these indices, the Root Mean Square Error of Approximation (RMSEA) was calculated at a 90% confidence interval, which must be ≤ .05 for a good fit, and from .05 to .08 for an acceptable fit. The Standardized Root Mean Square Residual (SRMR), which must be ≤ .05 for a good fit, and from .05 to .10 for an acceptable fit [57], was also calculated. Invariance of measurement of the SNS scale across sex and age was estimated. The sample was divided into two groups by age, from 11 to 15 years and from 16 to 18 years. We tested model fit separately for male and female and for younger and older adolescents and then a multigroup CFA was done. Configural invariance, in which the latent structure was constrained to be equal across groups (sex and age), was analyzed, and factor loadings and thresholds were estimated freely. After that, scalar invariance, in which the factor loadings and thresholds were constrained to be equal across sex and age was analyzed. Model fit was evaluated with the ΔCFI. There is invariance if the Δ in CFI is <.01 [58].

Reliability was analyzed with the ordinal alpha and McDonald’s Omega for the total scale. For evidence of convergent and discriminant validity, bivariate Spearman Correlation analyses were conducted. Finally, the ROC curve was calculated for SNS sensitivity and specificity. Statistical analyses were done with the SPSS, Lisrel 8.7, and Factor 10.5.03 programs.

## Results

### Descriptive analysis of the items on the SNS scale

The result of the Mardia’s test of multivariate asymmetry, skewness and kurtosis was 107.45 (*p* < .001). Table 1 shows the means, standard deviations, skewness, kurtosis and percentage of participants who answered the items affirmatively (Options 1 and 2). The items with the lowest percentage of positive answers were related to the social withdrawal factor (Items 3 and 4) and anhedonia (Items 17 and 18). Specifically, 86.7 and 74.6% answered Items 17 and 18 negatively, respectively. Skewness and kurtosis of items were below 2 and 7 respectively, expect for Item 3 (skewness = 2.78 and kurtosis = 7.08).

**Table 1:** Descriptive statistics of the items on the SNS scale

Items	*Mean*	*SD*	skewness	kurtosis	Percentage of affirmative responses^a^
1	.53	.63	.81	−.39	44.9
2	.31	.55	1.62	1.71	26.3
3	.17	.45	2.78	7.07	13.3
4	.31	.58	1.68	1.77	25.4
5	.51	.68	.99	−.25	40
6	.55	.68	.85	−.47	40
7	.56	.75	.92	−.62	40.3
8	.91	.76	.15	−1.27	66
9	.70	.74	.55	−1.01	52.9
10	.35	.63	1.56	1.18	27.1
11	.41	.67	1.36	.50	30.8
12	.59	.74	.83	−.71	43.7
13	.79	.71	.33	−1.01	61.9
14	.72	.70	.72	−.88	57.9
15	1.07	.77	1.07	−1.32	73.3
16	.62	.74	.62	−.84	45.9
17	.25	.53	.25	3.20	20.5
18	.27	.54	.27	2.64	22.2
19	.38	.61	1.37	.78	31.1
20	.61	.74	.77	−.81	45.4
Total	10.57	6.27	.73	.41	–

^a^Percentage of participants that marked response options 1 and 2

### Exploratory factor analysis

The EFA found adequate values in the KMO (.89, 95% CI = .881, .894) and Bartlett’s Sphericity (*χ*^2^_(190)_ = 14,040.9, *p* < .001) tests. Parallel analyses recommended a one-factor solution, however, the Schwarz’s Bayesian Information Criterion (BIC) and scree plot initially suggested a five-factor solution. These five factors coincide with the dimensions proposed by the authors of the scale and explained 57% of the variance. The factor loadings are shown in Table 2. The correlations between factors varied from *r* = .28 (Anhedonia-Avolition) to *r* = .55 (Alogia-Social withdrawal).

**Table 2:** Exploratory Factor Analysis rotated factor matrix loadings

	DER	AN	AL	AV	SW
Item 1	−.069	−.132	.044	.069	**.719**
Item 2	.029	.024	.042	.029	**.721**
Item 3	.133	.241	−.014	.011	**.489**
Item 4	.069	.259	−.034	−.009	**.476**
Item 5	**.452**	.035	.195	−.005	−.109
Item 6	**.556**	.031	−.009	.078	.050
Item 7	**.487**	.066	−.045	−.041	−.007
Item 8	**.327**	−.134	.232	.116	.134
Item 9	.218	−.031	**.316**	.079	.155
Item 10	−.021	−.017	**.790**	.060	.048
Item 11	.042	−.030	**.772**	−.072	−.025
Item 12	.006	−.081	**.320**	.289	.116
Item 13	−.095	−.082	.098	**.689**	−.013
Item 14	.022	.077	−.027	**.656**	−.023
Item 15	.080	−.091	.056	**.718**	.074
Item 16	.095	.058	−.013	**.490**	.047
Item 17	.096	**.339**	.220	−.009	.310
Item 18	.074	**.517**	.105	.223	.047
Item 19	.183	**.502**	.022	.120	−.005
Item 20	−.029	**.296**	.112	−.164	.059

*Note*. *DER* Diminished Emotional Range; *AN* Anhedonia, *AL* Alogia, *AV* Avolition, *SW* Social withdrawal

Primary loadings for each observed variable are in bold

### Confirmatory factor analysis

**Fig. 1:**
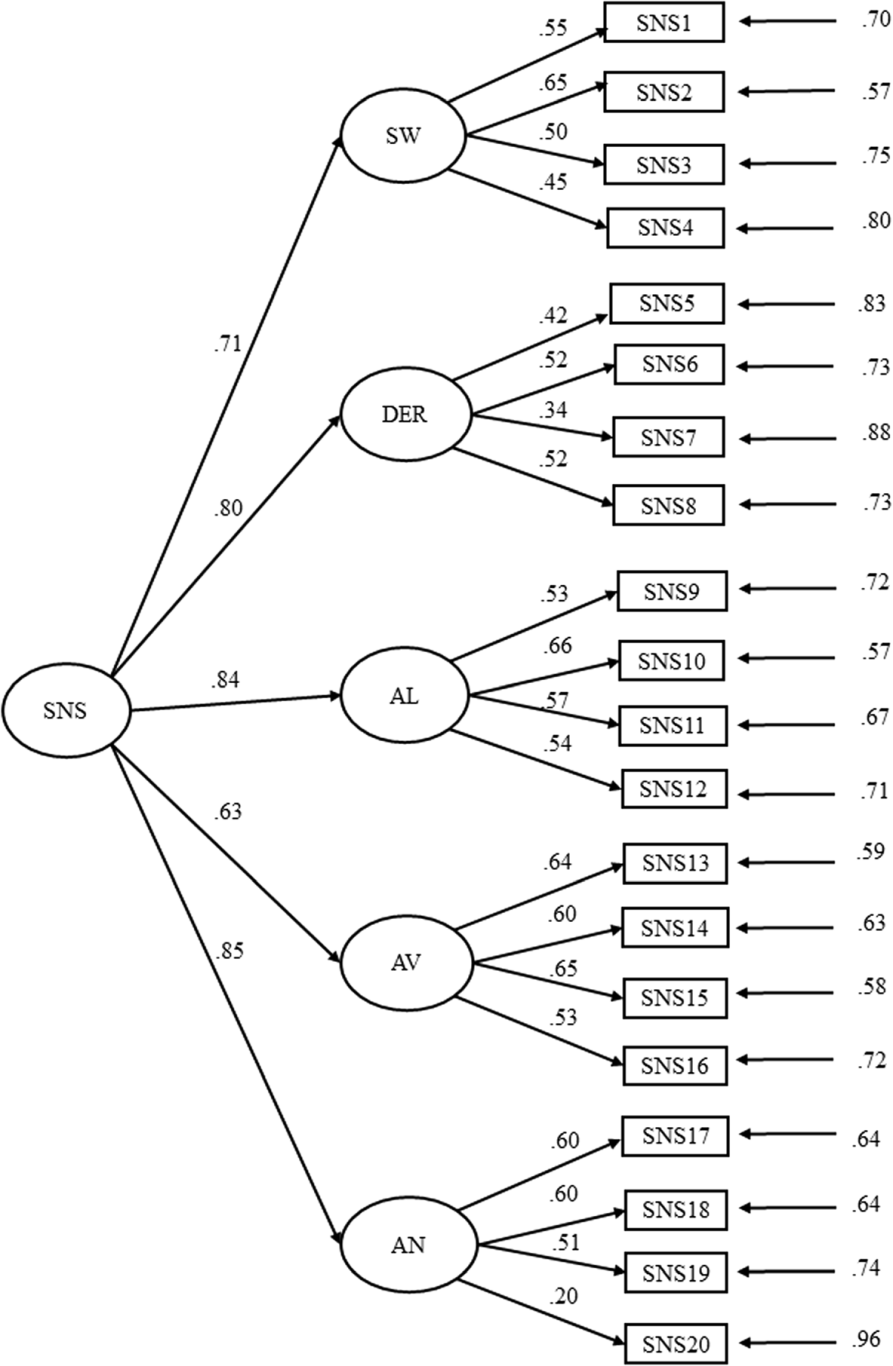
Path diagram and estimates for the five first-order factors related to a second-order of the SNS scale. *Note*. SW = Social withdrawal; DER = Diminished Emotional Range; *AL* Alogia, *AV* Avolition, *AN* Anhedonia

The CFA was performed with RDWLS estimation on the asymptotic covariance matrix. Four models were tested, the five factors found in the EFA (Model 1), the two factors found with patients by the authors of the scale (Model 2), a model with the five first-order factors and one second-order factor (Model 3), which would allow the SNS scale to be used by adding up its items to get a total score, and a unidimensional model following the recommendation of the parallel analyses. Table 3 shows the four models. All the models had adequate fit indicators. Figure 1 shows the completely standardized factor loadings for Model 3.

**Table 3:** Fit indices of the SNS scale

Model	*χ*^2^Satorra-Bentler	*df*	CFI	NNFI	SRMR	RMSEA [90% CI]	AIC
Model 1	1367.44	160	.983	.980	.049	.041 [.039, .043]	1466.79
Model 2	3242.57	169	.958	.953	.070	.063 [.062, .065]	3405.47
Model 3	1413.87	165	.983	.980	.045	.041 [.039, .043]	1503.73
Model 4	3369.12	170	.961	.957	.072	.065 [.063, .066]	3449.12

*Note*. Model 1: Five factors found from EFA, Model 2: Two factors proposed by the scale’s authors, Model 3: One second-order factor and five first-order factors found by EFA, Model 4: unidimensional model

In addition, to check whether the five-factor structure would be appropriate for participants with psychosis (schizophrenia), Model 3 was tested with subjects with a score > = to the 90th percentile on the SNS scale. The goodness-of-fit indicators were adequate: Satorra Bentler Chi^2^ = 267.33 (*df* = 165), RMSEA .035 [.027, .042], CFI = .92, NNFI = .90, SRMR = .076.

### Invariance of measurement across sex and age

An analysis of invariance of measurement was performed for sex and age. First the goodness-of-fit indicators for males and females were evaluated separately and then a multigroup CFA was done with the RDWLS method. The same was done for age. The results showed configural and scalar invariance of the model across sex and age (ΔCFI <.01), demonstrating that the factor structure of the SNS scale, the factor loadings and the thresholds are equivalent in males and females as well as ages (see Table 4).

**Table 4:** Multi-group CFAs testing for measurement invariance between sex and age SNS

	*χ*^2^Satorra-Bentler	*df*	CFI	NNFI	RMSEA [90% CI]	SRMR	ΔCFI
Sex							
Male (*n* = 2099)	571.13	165	.986	.984	.035 [.032, .038]	.048	
Female (*n* = 2422)	976.12	165	.981	.979	.045 [.032, .048]	.057	
Configural	1393.19	326	.984	.981	.038 [.036, .040]	.056	
Scalar	1820.44	375	.978	.978	.042 [.039, .043]	.068	−.006
Age							
Age:11–15 (*n* = 3301)	1003.96	165	.983	.980	.039 [.036, .042]	.049	
Age: 16–18 (*n* = 1220)	541.51	165	.983	.983	.043 [.039, .047]	.056	
Configural	1372.77	326	.984	.982	.038 [.036, .040]	.056	
Scalar	2103.62	375	.975	.974	.045 [.043, .047]	.073	−.009

### Reliability and evidence of validity of the SNS scale

The internal consistency of the SNS scale and subscales was estimated by finding the ordinal alpha coefficient on the polychoric correlations matrix and the McDonald’s Omega coefficient. The overall alpha for the complete SNS scale was .92, for social withdrawal it was .75, for avolition it was .76, for alogia .74, for anhedonia .61 and for diminished emotional range it was .59. The McDonald’s Omega coefficient was .87. The ASI, REF and CDI scales had favorable internal consistency (*α* ASI = .82, *α* REF = .82, *α* CDI = .83).

To study the evidence of convergent and divergent validity, the Spearman’s correlations were found between the total scores on the CDI depression scale, the REF for referential thinking, ASI for aberrant salience, and the SNS total score and factors. As shown in Table 5, all the correlations were statistically significant. The correlation results for the total SNS score and the CDI (*r* = .514) should be emphasized. The correlation between the ASI scale and the total SNS score was low, showing evidence of divergent validity, however, for the REF scale it was moderate.

**Table 5:** Spearman’s correlations between total scores on the REF Referential Thinking Scale, aberrant salience, CDI, SNS and avolition, social withdrawal, diminished emotional range, anhedonia and alogia subscales

	SNS total	AV	SW	DER	AN	AL
IR	.409**	.359**	.274**	.224**	.201**	.339**
ASI	.311**	.311**	.237**	.162**	.104**	.235**
CDI	.514**	.512**	.382**	.195**	.232**	.395**
*Mean*	10.57	3.23	1.32	2.49	1.48	2.04
*SD*	6.27	2.12	1.52	1.82	1.54	1.82

*Note*. *AV* Avolition, *SW* Social Withdrawal, *DER* Diminished Emotional Range, *AN* Anhedonia, *AL* Alogia, *IR* Ideas of reference, *ASI* Aberrant Salience, *CDI* Depression

** *p* < .01

The ROC curve was calculated to study the sensitivity and specificity of the SNS scale. The subjects in one group, with scores on the REF, ASI and CDI scales over the 85th percentile, were identified as being at risk (*n* = 81), and the rest of the sample was in another. The ROC curve showed a significant area of .81 [95% CI = .773–.853] for a cutoff point of 13.5 points, sensitivity of .73 and specificity of .72. With the sample percentile applied to the SNS scale (85th percentile, starting from 17 points), 16.96% of the participants were found to have high NS scores (51.4% males).

## Discussion

Early identification of psychosis is a priority which its development and consequent functional impairment depend on, in addition to personal, family and healthcare costs [59]. Much of the research has concentrated on the population that has requested clinical help, and mainly, with risk criteria based on positive symptomatology. However, negative/disorganized symptoms are those that predict positive symptoms (and not the other way around), and persistence of the first are a key element in prediction [29]. Therefore, study of NS such as Psychotic-Like Experiences (PLEs) during adolescence could pose an advantage for properly characterizing the complex process that could culminate in development of psychosis.

The presence of NS in the general population and in young people at clinical risk [7, 60] requires simple, brief measures which facilitate their detection. The main objective of this study was to adapt and validate the SNS to Spanish. This would enable its application in a general adolescent population to identify self-informed NS for the broad Spanish-speaking context.

Although it is not expected to find a high frequency of NS in the general adolescent population not requesting clinical help [26], it is true that their presence may be demonstrated. This study found that from 13.3 to 73.3% of adolescents showed some NS on the SNS scale, and almost 17% had high scores (85th percentile). These results are near those found by Werbeloff et al. [17].

Therefore, although NS are relatively frequent in the general population, they are mostly of lower intensity, and probably transitory [20, 61, 62], which would fit in with the PLE concept. A cut-off point of 13 was found on the SNS, with lower sensitivity and specificity indicators than those reported by the instrument’s authors, probably because our study focused on the general population. Although this population is known not to usually require medical attention [25], risk of psychosis is not discarded [63, 64]. Social withdrawal, and in second place, anhedonia, were the least outstanding NS. This was expected since lack of interest and disconnection with others are characteristic indicators of schizophrenia, risk factors with predictive power for psychosis [34, 65]. The outstanding factor at any intensity was motivational (avolition), perhaps reflecting the changes the adolescent has to cope with until achieving self-regulation: between exploration and adjustment to external and internal demands [66].

The second objective of this study focused on determining the factorial structure of the SNS scale. Thus, it could be shown whether the NS observed in an adolescent general population represent constructs similar to those found in other studies, usually with patients. The structure of the SNS scale (EFA) showed the factors proposed by the authors [11] and agreed with NIMH and MATRICS: alogia, social withdrawal, anhedonia, diminished emotional range and avolition, even though parallel analysis recommended a unidimensional structure. The five-factor structure was confirmed (CFA) with adequate indicators and more parsimoniously than the two avolition/apathy/amotivation and expressive factors observed in patients with schizophrenia [9, 11]. Because of the recommendation of the parallel analyses to consider the SNS unidimensional, it was decided to try a second-order model providing a total score in negative symptomatology which could be used as a criterion of clinical severity. Considering that a second-order model is more restrictive than a first-order one and that, in spite of it, Model 3 has a parsimonious fit, we think it is of interest to go with this model.

Summarizing, although generally and with an adolescent population, the characteristics observed by the authors of the SNS in an adult population, mainly patients, were maintained with an internal structure that characterizes general affective-motivational responses, where the severity with which its absence is observed is relevant in determining the NS, at least as a criterion of study when applied to a general population like a PLE.

The third objective of this study concentrated on the way in which adolescents responded to the SNS scale. The analysis of invariance across sex and age showed that the SNS can be used without these variables influencing how the instrument is answered. This result is relevant considering that adolescents from 11 years of age participated, and was therefore shown to be an adaptation of this scale adequate for its use in this population.

The following objective of the analysis of the psychometric properties of the SNS showed that the reliability of the overall scale was favorable. Internal consistency was adequate for social withdrawal, alogia and avolition, but unfavorable for anhedonia and diminished emotional range. In the case of anhedonia, Item 20 (interest in sex), was problematic, above all among the youngest, and Items 17 and 18 had a very low response. However, what was on target in this factor was that Item 19 was related to anticipatory pleasure. The internal consistency of anhedonia in general could have been problematic, because its independence from depressive symptomatology was not clear with the design applied [67]. It is also possible that stress attenuates the reward system response [68] precisely when studied in youths in the general population. Furthermore, the reliability found in the diminished emotional range factor could be affected by defining indicators that require either observation by others (Item 5 considers the point of view of others) or good capacity for self-evaluation by the adolescent.

In divergent validity, moderate-to-low correlation with indicators representative of positive symptomatology (ideas of reference and aberrant salience) shows that these symptoms are different from NS. A low but not absent relationship suggests the need to verify, as pointed out by Jones et al. [63], whether NS and anxiety can cause errors in processing attributional salience of anomalies.

The high correlation with depressive symptomatology does not clarify differentiation between NS and depressive symptoms [69]. The factor most closely related to depressive symptoms was avolition, and the one which was the least related was diminished emotional range, as would be expected from its specificity to the psychotic scope. The difficulty in differentiating NS from depressive symptoms and whether NS are secondary to depression, cannot be elucidated with this design [34].

Some suggestions coming out of this study are that the low indices for prediction of the transition to psychosis are due precisely to not taking the mood symptomology into account in its UHR criteria [42, 43]. In like manner, it is probable that the prodromal indicators should be considered pleiotropic because they lead to psychotic and nonpsychotic manifestations (such as depression, anxiety or substance use), so in the evaluation of PLEs, overlapping can be observed which does not specify the final trajectory until its persistence and combination with other variables open the way for psychotic development [70].

The contributions of this study have limitations which should be taken into account. It is a cross-sectional study with the drawbacks typical of a single evaluation. The adolescents considered at risk were part of a clinical evaluation and follow-up, results of which, for reasons of space, were not included in this article. This design conditions being able to determine NS as PLE which could later cause full psychotic development. A prospective design would be required to find out the true predictive capacity of the NS as PLE. It should also be kept in mind that indicators of severity were considered to conceive psychometric risk, but they were based on a normal population. This decision is not exempt from drawbacks, but for the purpose of screening, it may still be useful, as long as it is corroborated with other measures or exhaustive clinical interview.

It is still important to analyze the persistence of NS and their genetic-environmental context [28, 71] and premorbid adjustment, to clarify whether NS are schizotypal/schizoid traits [46, 47] and compare a group of young people with a first episode of psychosis to the results with the general population, limiting their generalization and applicability. Furthermore, it would have to be demonstrated that the stability of the measure is reliable. However, replication of the same NS structure with a general and adolescent population could be an indirect indicator that this construct is stable, possibly as a trait. Nevertheless, the last of the objectives was to verify adequate indicators of sensitivity and specificity for an established risk criterion, and this was done. This said, these indicators must be taken with caution in the context of a first evaluation or screening and not as an established risk or with diagnostic characteristics.

Strauss and Gold [72] emphasized that one of the disadvantages of self-reports is that they lead to more semantic processing than experiential (e.g., evaluating beliefs about pleasure). In particular, evaluation of the emotional range/intensity requires observation more than subjective appreciation [5], decreased spontaneous movement being a key indicator of NS severity [73] which cannot be captured in a self-report. The presence of false positives is also expectable with self-report measures, but as suggested by Kaymaz [74], risk is not discarded because of false positives, especially, with high scores.

Another difficulty was lacking another NS scale to calculate the convergent validity. However, its inclusion would have lengthened testing, reducing reliability from tiring during its collective application.

In spite of its limitations, this study offers an outstanding contribution in the scope of evaluation of NS in adolescents, by facilitating the communication of relevant and complex inner experiences [3], in view of the shortage of self-report instruments specific to NS.

## Conclusion

The SNS scale is measure that could be used for screening in academic orientation and in healthcare because of its brief application and simple items. Later evaluation by the specialist and information from parents and teachers can culminate in better accuracy in identification and follow-up of NS.

## Data Availability

The datasets generated and/or analysed during the current study are available in the figshare repository, https://figshare.com/s/ca65a594d4ccfab10bb6

## Abbreviations

AL: Alogia
AN: anhedonia
ASI: Aberrant Salience Inventory
AV: Avolition
BIC: Schwarz’s Bayesian Information Criterion
CDI: Children’s Depression Inventory
CFA: Confirmatory Factor Analysis
CFI: Comparative Fit Index
DER: Diminished Emotional Range
EFA: Exploratory Factor Analyses
GFI: Goodness of Fit Index
KMO: Kaiser-Meyer-Olkin
MATRICS: Measurement and Treatment Research to Improve Cognition in Schizophrenia
NIMH: National Institute of Mental Health
NNFI: Non-Normed Fit Index
NS: Negative symptoms
PLE: Psychotic-Like Experiences
RDWLS: Robust Diagonally Weighted Least Squares
REF: Referential Thinking Scale
RMSEA: Root Mean Square Error of Approximation
SCI: Social Class Index
SNS: Self-Evaluation of Negative Symptoms Scale
SRMR: Standardized Root Mean Square Residual
SW: Social withdrawal
UHR: Ultra High Risk
